# Combination of Near-Infrared Photoimmunotherapy Using Trastuzumab and Small Protein Mimetic for HER2-Positive Breast Cancer

**DOI:** 10.3390/ijms222212213

**Published:** 2021-11-11

**Authors:** Haruka Yamaguchi, Jotaro On, Takao Morita, Takamasa Suzuki, Yasuo Okada, Junya Ono, Andreas Evdokiou

**Affiliations:** 1 Department of Biochemistry, School of Life Dentistry at Niigata, The Nippon Dental University, Niigata 951-8580, Japan; harukay@ngt.ndu.ac.jp (H.Y.); moritat@ngt.ndu.ac.jp (T.M.); 2 Discipline of Surgery, Breast Cancer Research Unit, Basil Hetzel Institute, University of Adelaide, Adelaide, SA 5011, Australia; 3 Department of Neurosurgery, Brain Research Institute, Niigata University, Niigata 951-8122, Japan; jotaro-on_silver@sky.hi-ho.ne.jp; 4 Faculty of Engineering, Niigata University, Niigata 950-2181, Japan; takamasa@eng.niigata-u.ac.jp; 5 Department of Pathology, School of Life Dentistry at Niigata, The Nippon Dental University, Niigata 951-8580, Japan; yokada@ngt.ndu.ac.jp (Y.O.); johno@ngt.ndu.ac.jp (J.O.)

**Keywords:** photoimmunotherapy, Affibody, trastuzumab, IR700Dye

## Abstract

Near-infrared photoimmunotherapy (NIR-PIT) is a promising cancer therapy based on a monoclonal antibody conjugated to a photosensitizer (IR700Dye) that is activated by near-infrared light irradiation. We previously reported on the use of NIR-PIT with a small protein mimetic, the Affibody molecule (6–7 kDa), instead of a monoclonal antibody. In this study, we investigated a combination of NIR-PIT for HER2-positive breast cancer cells (SK-BR3, MDA-MB361, and JIMT1) with HER2 Affibody-IR700Dye conjugate and trastuzumab-IR700Dye conjugate. HER2 Affibody and trastuzumab target different epitopes of the HER2 protein and do not compete. In vitro, the combination of NIR-PIT using both HER2 Affibody-IR700Dye conjugate and trastuzumab-IR700Dye conjugate induced necrotic cell death of HER2-positive breast cancer cells without damage to HER2-negative breast cancer cells (MCF7). It was more efficient than NIR-PIT using either the HER2 Affibody-IR700Dye conjugate alone or the trastuzumab-IR700Dye conjugate alone. Additionally, this combination of NIR-PIT was significantly effective against HER2 low-expressing cancer cells, trastuzumab-resistant cells (JIMT1), and brain metastatic cells of breast cancer (MDA-MB361). Furthermore, in vivo imaging exhibited the strong fluorescence intensity of both HER2 Affibody-IR700Dye conjugates and trastuzumab-Alexa488 conjugates in HER2-positive tumor, indicating that both HER2 Affibody and trastuzumab specifically bind to HER2-positive tumors without competing with each other. In conclusion, the combination of NIR-PIT using both HER2 Affibody and trastuzumab expands the targeting scope of NIR-PIT for HER2-positive breast cancer.

## 1. Introduction

In this study, we targeted HER2-positive breast cancer because HER2-positive expression occurs in about 20% of patients with breast cancer and is generally linked to poor outcomes [1,2]. The HER2 protein is a 185-kilodalton transmembrane receptor and belongs to the tyrosine kinase epidermal growth factor receptor family, which promotes cell growth, division, and motility. Compared to other subtypes, HER2-positive cancers grow faster due to increased HER2 signaling. Therefore, there are many studies on cancer treatment that target the HER2 protein [3,4,5]. However, cancers are heterogeneous, and HER2-positive cancer also includes cancer cells with low HER2 expression. In addition, a significant number of patients with HER2-positive cancer are either intrinsically resistant or eventually acquire resistance to anti-HER2-based therapy with trastuzumab, which is a major monoclonal antibody used to clinically treat HER2-positive cancer [6]. Therefore, there is an urgent need for new treatment to break away from the current state of HER2-positive cancer therapy.

Near-infrared photoimmunotherapy (NIR-PIT) is a promising cancer therapy using a monoclonal antibody-conjugated photosensitizer (IR700Dye) and near-infrared light, causing necrotic cancer cell death without an effect on normal cells [7,8,9]. In NIR-PIT, monoclonal antibody (mAb)-conjugated IR700Dye binds to a specific protein on the target cell surface, and when near-infrared light (690 nm) is irradiated, the light excites the IR700Dye, causing damage to the cell membrane [10]. Currently, NIR-PIT targeting EGFR with an mAb-IR700Dye conjugate is under a global phase III clinical evaluation for the treatment of head and neck cancer (NCT03769506).

We previously reported on the use of NIR-PIT with a small protein mimetic, the Affibody molecule (6–7 kDa), instead of a monoclonal antibody. Affibody molecules are derived from the B-domain in the immunoglobulin-binding region of staphylococcal protein A to recognize various molecules [11,12,13] and are used for imaging and therapy [14,15]. Due to their small size, NIR-PIT using Affibody molecules may expand the targeting scope of NIR-PIT for HER2-positive breast cancer [16]. However, the effect of NIR-PIT depends on the level of expression of the targeted protein. When the targeted protein expression is low, IR700Dye cannot be activated efficiently, leading to reduced cellular damage and, therefore, the likelihood of cancer recurrence.

In this study, we investigated the combination of NIR-PIT using a HER2 Affibody-IR700Dye conjugate and a trastuzumab-IR700Dye conjugate which target different epitopes of HER2 protein. Here, we demonstrated that the combination of NIR-PIT potentiates the effect of NIR-PIT against HER2-positive breast cancer cells, including breast cancer cells with low HER2 expression, trastuzumab-resistant breast cancer cells [17], and brain metastatic breast cancer cells. It can be a new strategy to treat HER2-positive breast cancer.

## 2. Results

### 2.1. Expression of Human Epidermal Growth Factor Receptor 2 (HER2)

**Figure 1:**
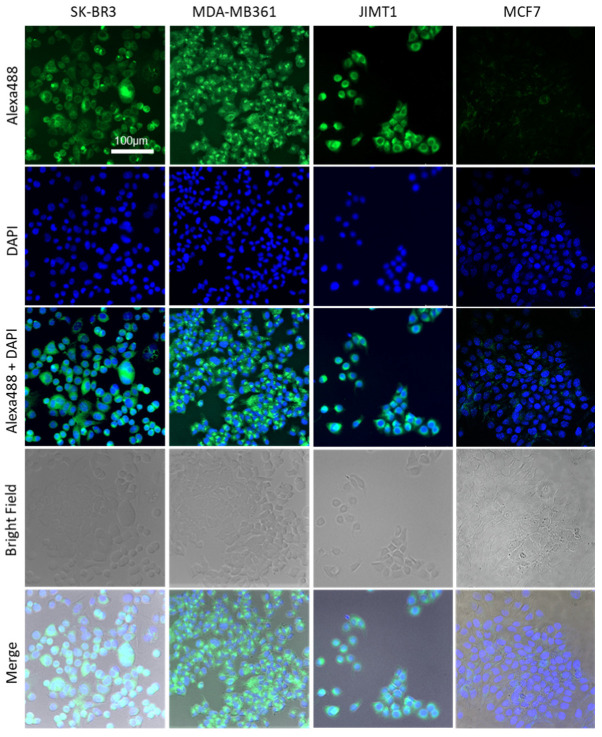
Immunocytochemistry of HER2 protein in breast cancer cell lines (SK-BR3, MDA-MB361, JIMT1, and MCF7). SK-BR3 cells, MDA-MB361 cells, and JIMT1 cells exhibited stronger fluorescent signals than MCF7 cells, in which HER2 expression was virtually undetected. Scale bar: 100 µm.

**Figure 2:**
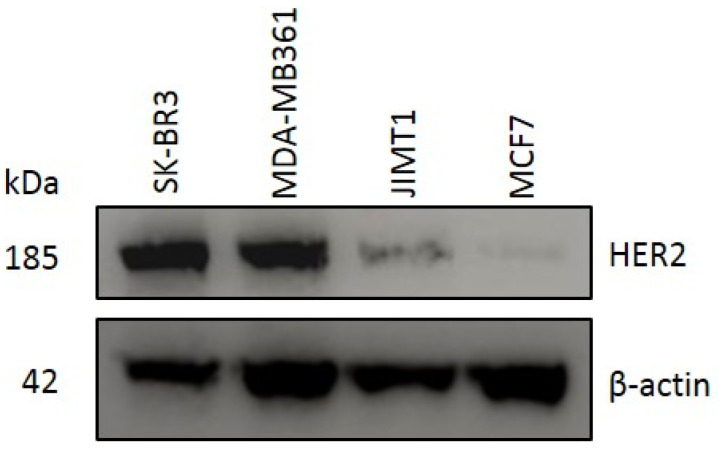
Western blotting analysis of HER2 protein in breast cancer cell lines (SK-BR3, MDA-MB361, JIMT1, and MCF7). β-actin protein expression was assessed as a control. A strong band with a molecular weight of 185 kDa, corresponding to HER2 protein, was observed in SK-BR3, MDA-MB361, and JIMT1 cells.

Various breast cancer cell lines were investigated for human epidermal growth factor receptor 2 (HER2) expression by immunocytochemistry (ICC) and Western blotting analysis. In ICC, SK-BR3 cells, MDA-MB361 cells, and JIMT1 cells showed stronger fluorescent signals than MCF7 cells, in which HER2 expression was virtually undetected (Figure 1). In the Western blotting analysis, a strong band with a molecular weight of 185 kDa corresponding to the HER2 protein was observed in SK-BR3, MDA-MB361, and JIMT1 cells. JIMT1 cells showed a weaker band than SK-BR3 and MDA-MB361 cells (Figure 2).

### 2.2. Localization of HER2 Affibody-IR700Dye and Trastuzumab-Alexa488 Conjugates

**Figure 3:**
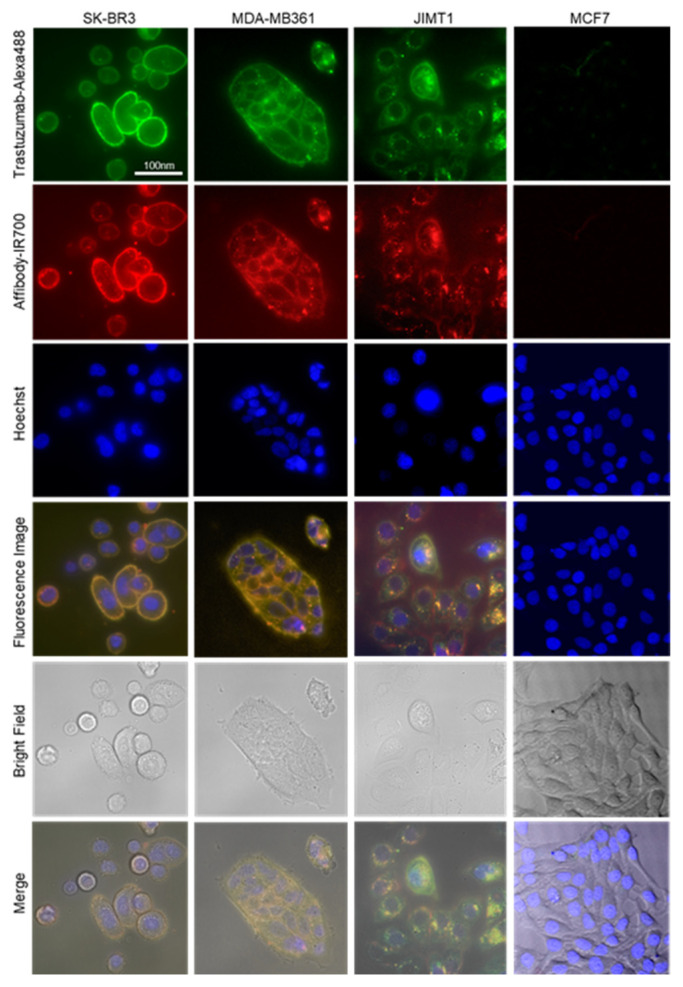
Fluorescence images of the cells show HER2-specific localization of HER2 Affibody-IR700Dye conjugate and trastuzumab-Alexa488 conjugate in HER2-positive cells (SK-BR3, MDA-MB361, and JIMT1) but not HER2-negative cells (MCF7).

**Figure 4:**
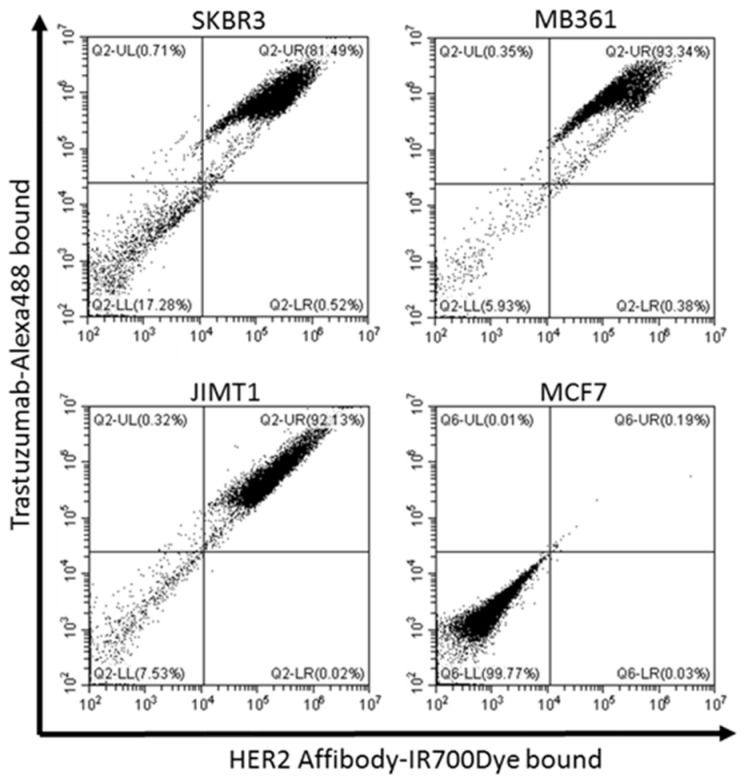
Flow cytometry after double labeling of HER2 Affibody-IR700Dye conjugate and trastuzumab-Alexa488 conjugate to HER2 receptors. Both IR700Dye and Alexa488 showed a strong fluorescence intensity and revealed specific binding of HER2 receptors on HER2-positive cells (SK-BR3, MDA-MB361, and JIMT1) without labeling to HER2-negative cells (MCF7). Representatives of each experiment are shown by dot plots.

To detect the HER2-specific localization of the HER2 Affibody-IR700Dye conjugate and the trastuzumab-Alexa488 conjugate, fluorescence images were taken using fluorescence microscopy (BZ-X800, KEYENCE, Osaka, Japan). The fluorescence of Alexa488 and IR700Dye was detected primarily on the cell surface of HER2-positive cells (SK-BR3, MDA-MB361, and JIMT1), while HER2-negative cells (MCF7) did not show any detectable fluorescence under the same imaging conditions (Figure 3). Furthermore, the flow cytometry analysis revealed the stronger fluorescence intensity of both Alexa488 and IR700Dye in the SK-BR3, MDA-MB361, and JIMT1 cell populations than in the MCF7 cell population (Figure 4).

### 2.3. The Morphological Effect by NIR-PIT

The images of the cancer cells showed that the HER2-positive cells (SK-BR3, MDA-MB361, and JIMT1) exposed to NIR-PIT with the HER2 Affibody-IR700Dye conjugate or the trastuzumab-IR700Dye conjugate displayed morphological evidence of cellular bursting and bleb formation, whereas the morphology of HER2-negative cancer cells (MCF7) remained unchanged (Supplementary Figure S1). Furthermore, as shown in Supplementary Figure S2, in the apoptosis/necrosis assay, HER2-positive cells exposed to NIR-PIT with the HER2 Affibody-IR700Dye conjugate or the trastuzumab-IR700Dye conjugate showed red signals, indicative of necrotic dead cells, while HER2-negative cells were stained blue, indicative of living cells, showing that the mode of death by NIR-PIT using the HER2 Affibody-IR700Dye conjugate or the trastuzumab-IR700Dye conjugate for HER2-positive cells is selectively necrotic.

### 2.4. Cell Viability after Near-Infrared Photoimmunotherapy (NIR-PIT)

**Figure 5:**
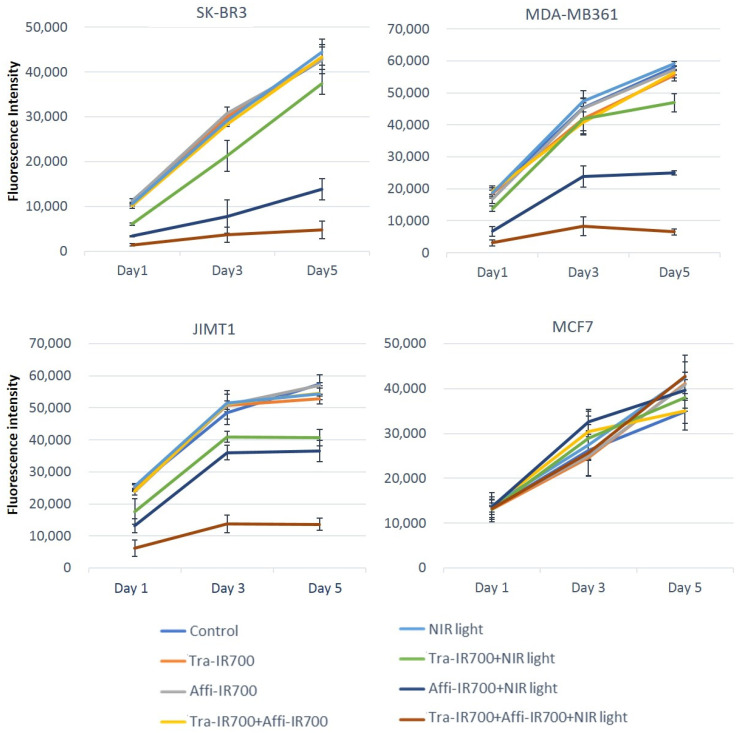
alamarBlue assay showed cell viability as fluorescence intensity. After NIR-PIT using HER2 Affibody-IR700Dye conjugate and/or trastuzumab-IR700Dye conjugate, the cell viability of SK-BR3 (0–0.1 µM, 0–5 J/cm^2^), MDA-MB361 (0–0.2 µM, 0–20 J/cm^2^), JIMT1 (0–0.3 µM, 0–50 J/cm^2^), and MCF7 (0–0.3 µM, 0–50 J/cm^2^) cells was measured for an extended period of 5 days. Only HER2-positive cells (SK-BR3, MDA-MB361, and JIMT1) treated with the combination of NIR-PIT with HER2 Affibody-IR700Dye conjugate and trastuzumab-IR700Dye conjugate maintained the lowest cell viability, whereas all samples of HER2-negative cells (MCF7) increased at the same speed as the control.

**Figure 6:**
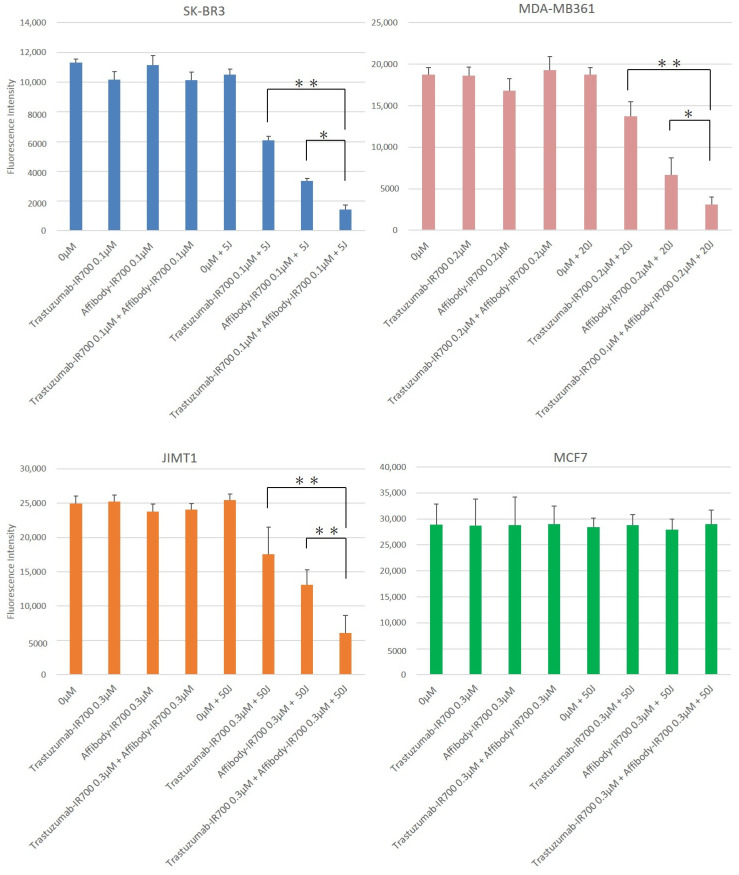
Fluorescence intensity indicating the cell viability 24 h after NIR-PIT. HER2-positive cells (SK-BR3, MDA-MB361, and JIMT1) treated with the combination of NIR-PIT showed lower cell viability than the cells treated with NIR-PIT using either HER2 Affibody-IR700Dye conjugate or trastuzumab-IR700Dye conjugate, with no effect of NIR-PIT on MCF7 cells. When SK-BR3 cells were exposed to 0.1 µM of the conjugates and 5 J/cm^2^ irradiation, MDA-MB361 cells were exposed to 0.2 µM of the conjugates and 20 J/cm^2^ irradiation, and JIMT1 cells were exposed to 0.3 µM of the conjugates and 50 J/cm^2^ irradiation, they showed the most significant difference between NIR-PIT using either HER2 Affibody-IR700Dye conjugate or trastuzumab-IR700Dye conjugate and the combination of both with NIR-PIT (*n* ≥ 6; * *p* < 0.05; ** *p* < 0.01, one-way ANOVA with Tukey–Kramer post hoc tests). Data are presented as means ± SD.

The alamarBlue assay showed cell viability as fluorescence intensity. As shown in Figure 5, HER2-positive cells (SK-BR3, MDA-MB361, and JIMT1) incubated with the HER2 Affibody-IR700Dye conjugate and the trastuzumab-IR700Dye conjugate (0.1–0.3 µM) and irradiated with NIR light (5–50 J/cm^2^) maintained lower cell viability than the cells treated with NIR-PIT using either the HER2 Affibody-IR700Dye conjugate alone or the trastuzumab-IR700Dye conjugate alone, even 5 days after irradiation. On the other hand, all samples of HER2-negative cells (MCF7) increased rapidly, including the cells that were exposed to the conjugates (0.3 µM each) and irradiated with NIR light (50 J/cm^2^) (Figure 5). Twenty-four hours after NIR-PIT, the viability of SK-BR3 (0.1 µM, 5 J/cm^2^), MDA-MB361 (0.2 µM, 20 J/cm^2^), and JIMT1 (0.3 µM, 50 J/cm^2^) cells showed a significant difference between NIR-PIT using either the HER2 Affibody-IR700Dye conjugate alone or the trastuzumab-IR700Dye conjugate alone and the NIR-PIT combination (Figure 6). On the contrary, all samples of MCF7 cells increased at the same level as control cells.

However, as shown in Figure 6, while SK-BR3 cells treated with the combination of NIR-PIT with the HER2 Affibody-IR700Dye and the trastuzumab-IR700Dye (0.1 µM, 5 J/cm^2^) conjugates were efficiently affected, MDA-MB361 cells and JIMT1 cells were unaffected under the same condition (Supplementary Figure S3). In addition, JIMT1 cells were not affected even by the NIR-PIT combination when the concentration of both conjugates was 0.2 µM and the power of irradiation was 20 J/cm^2^, while MDA-MB361 cells were affected (Figure 3). Taken together, the cell viability of HER2-positive cells depends on the level of HER2 expression, the concentration of the conjugates, and the dose of NIR light irradiation.

### 2.5. In Vivo Fluorescence Image

**Figure 7:**
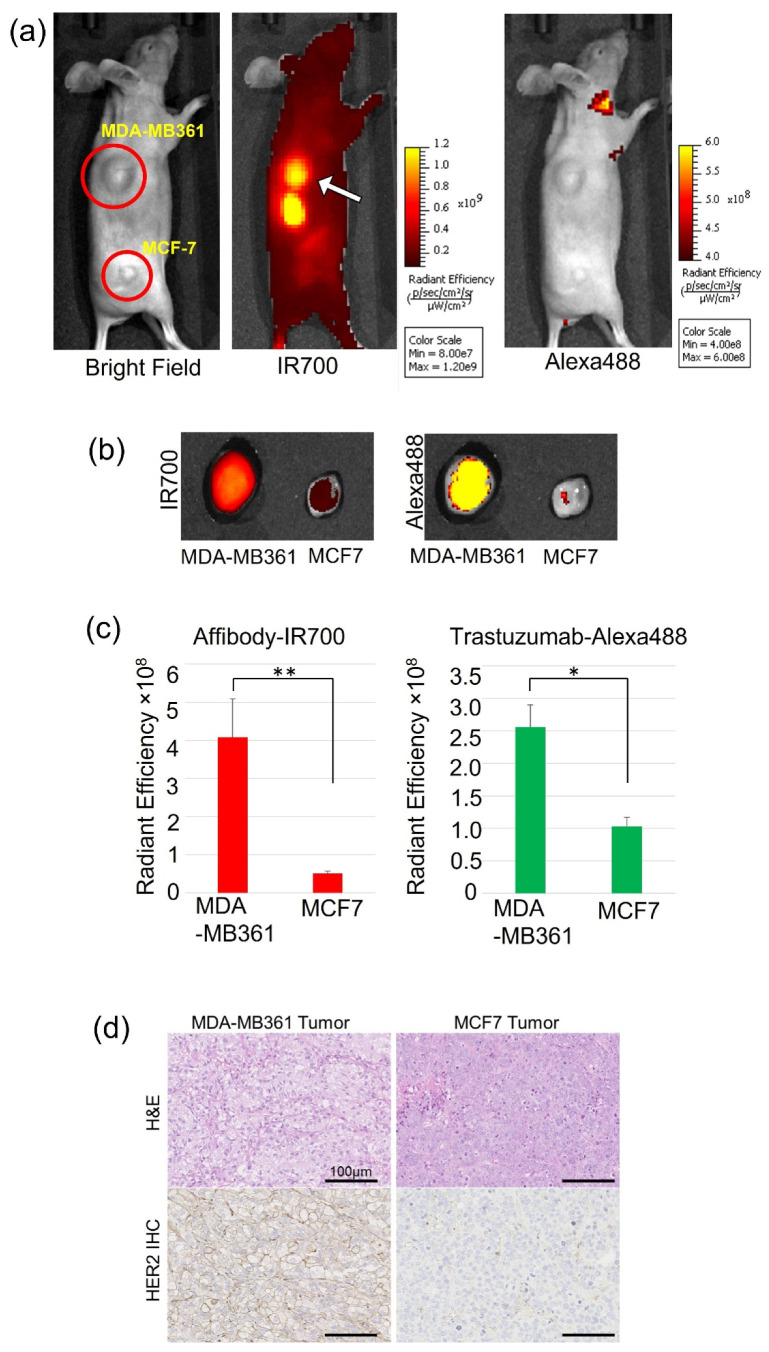
(**a**) In vivo imaging of tumor xenograft-bearing mice with HER2 Affibody-IR700Dye conjugate and trastuzumab-Alexa488 conjugate. The image shows high-intensity IR700Dye fluorescence in the MDA-MB361 tumor (shoulder, white arrow) in contrast to the MCF7 tumor (right dorsum). (**b**) The excised MDA-MB361 tumor exhibited higher fluorescence intensity of HER2 Affibody-IR700Dye conjugate and trastuzumab-Alexa488 conjugate in contrast to the MCF7 tumor. (**c**) The fluorescence intensities of IR700Dye and Alexa488 showed a significant difference (*n* = 3, * *p* < 0.05; ** *p* < 0.01, Student’s *t*-test). Data are presented as means ± SD. (**d**) The immunohistochemistry of HER2 protein from the engrafted tumors revealed stronger positive HER2 staining on the cell membrane of the MDA-MB361 tumor than on the MCF7 tumor.

In the mouse tumor xenograft studies, the mouse bearing both types of tumors exemplified the difference between the two tumors (Figure 7a, white arrow). The MDA-MB361 tumor had a stronger fluorescence intensity of IR700Dye than the MCF7 tumor did, while Alexa488 was not detectable in both tumors. In the images of excised tumors (the weight (mean ± SD) of MDA-MB361 (*n* = 3) was 0.36 ± 0.121 g and that of MCF7 (*n* = 3) was 0.046 ± 0.012 g) in Figure 7b, the MDA-MB361 tumor exhibited a stronger fluorescence intensity of both IR700Dye and Alexa488 than the MCF7 tumor did, and the fluorescence intensity showed significant differences (Figure 7c). The MCF7 tumor grew slower than the MDA-MB361 tumor, but the MCF7 tumor had enough vascularization to be affected by the conjugates. Moreover, HER2 immunohistochemistry of engrafted tumors revealed stronger HER2 positive stains in the MDA-MB361 tumor than in the MCF7 tumor (Figure 7d).

**Figure 8:**
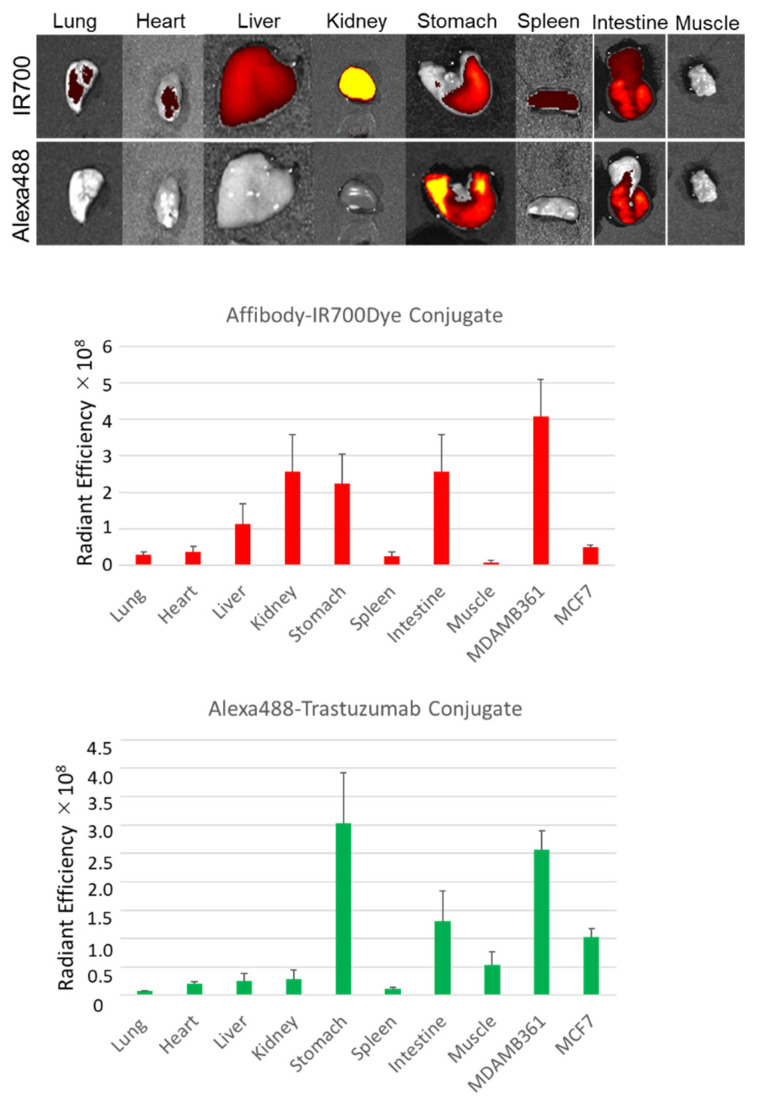
Fluorescence intensity from the individual organs of the mice injected with HER2 Affibody-IR700Dye conjugate and trastuzumab-Alexa488 conjugate. Data are presented as means ± SD (*n* = 3).

Next, the fluorescence intensities from the tissues were examined ex vivo. The fluorescence images of IR700Dye showed strong fluorescence intensity in the liver, kidney, stomach, and intestine. On the other hand, the fluorescence images of Alexa488 showed strong fluorescence intensity only in the stomach and the intestine (Figure 8).

## 3. Discussion

To determine the effect of the combination of near-infrared photoimmunotherapy (NIR-PIT) with the HER2 Affibody-IR700Dye conjugate and the trastuzumab-IR700Dye conjugate, we performed a range of analyses. Our immunocytochemistry (ICC) and Western blotting analyses demonstrated strong expression of the HER2 protein in SK-BR3, MDA-MB361, and JIMT1 cancer cells compared to MCF7 cancer cells (Figure 1 and Figure 2). JIMT1 cells expressed the HER2 protein less than SK-BR3 and MDA-MB361 cells did (Figure 2). These results are in line with those reported by others in the literature [7,8]. In Figure 3, the fluorescence images show that IR700Dye and Alexa488 from the HER2 Affibody-IR700Dye conjugate and the trastuzumab-Alexa488 conjugate clearly merged in the HER2-positive cancer cells. Additionally, in Figure 4, flow cytometry, used to measure the fluorescence intensity on the surface of cells in a population, showed that the fluorescence intensity of IR700Dye and Alexa488 in the HER2-positive cells was stronger than in the HER2-negative cells after double labeling of the HER2 Affibody-IR700Dye conjugate and the trastuzumab-Alexa488 conjugate. These results indicate that the HER2 Affibody-IR700Dye conjugate and the trastuzumab-Alexa488 conjugate do not compete with each other and are specifically bound to the HER2 protein (Figure 3 and Figure 4). In Supplementary Figures S1 and S2, our results show that NIR-PIT using either the HER2 Affibody-IR700Dye conjugate or the trastuzumab-IR700Dye conjugate induced morphological changes of bursting as well as necrotic cell death of only HER2-positive cancer cells without any damage to HER2-negative cancer cells. Sato et al. described that NIR-PIT using mAbs induces physical changes in the conjugate that is bound to the surface of target cells, exerting physical stress within the cellular membrane and leading to an overall increase in transmembrane water flow that eventually leads to cell bursting and necrotic cell death [18]. NIR-PIT using either the HER2 Affibody-IR700Dye conjugate or the trastuzumab-IR700Dye conjugate also induced the same physical changes against HER2-positive cancer cells.

Our combination of NIR-PIT using the HER2 Affibody-IR700Dye conjugate and the trastuzumab-IR700Dye conjugate caused selective cell death of HER2-positive cancer cells and successfully maintained the lowest cell viability even 5 days after the NIR-PIT (Figure 5 and Figure 6 and Supplementary Figure S3).

Affibody molecules bind to an epitope that includes residues from both domains III and Ⅳ of the HER2 protein, which are different from the epitopes where trastuzumab binds [19]. Using the difference of the binding epitopes, Lee et al. successfully exhibited fluorescence images of HER2-positive cells with double labeling of Affibody-Alexa488 and trastuzumab-Alexa680 conjugates [20]. Additionally, previous studies investigated the efficacy of NIR-PIT using a cocktail of antibody conjugates and concluded that it enhanced the therapeutic effects of NIR-PIT [21,22]. In this study, the difference of the targeted epitope of the HER2 protein between HER2 Affibody and trastuzumab facilitated cooperation and enhanced the effect of NIR-PIT on HER2-positive cancer cells.

Our results clearly indicated that the effect of NIR-PIT on cells was well correlated with the level of HER2 protein expression on the targeted cells, the concentration of the conjugate, and the power of irradiation (Figure 5 and Figure 6 and Supplementary Figure S3). SK-BR3 cells expressed adequate HER2 protein to be efficiently killed by NIR-PIT using either the HER2 Affibody-IR700Dye conjugate alone or the trastuzumab-IR700Dye conjugate alone when the concentration of the conjugates was high (0.2–0.3 µM) and the power of irradiation was strong (20–50 J/cm^2^). However, JIMT1 cells were not affected even by the NIR-PIT combination using both conjugates (0.2 µM) and NIR light irradiation (20 J/cm^2^) (Supplementary Figure S3). This is because the expression of the HER2 protein in JIMT1 is low, and also because JIMT1 has a low level of trastuzumab binding capacity to HER2 receptors despite amplification of the HER2 gene [23]. JIMT1 is a cancer cell line that was established from a pleural metastasis of a patient with breast cancer who was clinically resistant to trastuzumab [17,24,25,26]. Importantly, the combination of NIR-PIT using both the HER2 Affibody-IR700Dye conjugate and the trastuzumab-IR700Dye conjugate (0.3 µM, irradiation: 50 J/cm^2^) successfully killed JIMT1 cells by about 80% without damaging HER2-negative cells (MCF7) (Figure 6). Our study is the first report to show that the combination of NIR-PIT is available to cancer cells that have acquired resistance to trastuzumab.

MDA-MB361 cells, which were isolated from a metastatic site in the brain, were also markedly affected by the combination of NIR-PIT, although our previous study showed that the cell viability slightly recovered 5 days after NIR-PIT using the HER2 Affibody-IR700Dye conjugate alone [16]. The combination of NIR-PIT using the HER2 Affibody-IR700Dye conjugate and the trastuzumab-IR700Dye conjugate has real therapeutic potential for brain metastases of HER2-positive cells because when cancer metastasizes to the brain, the blood–brain barrier (BBB) is disrupted [27], consequently permitting both monoclonal antibodies and Affibody molecules to cross [28,29,30,31].

Taken together, our combination of NIR-PIT using the HER2 Affibody-IR700Dye conjugate and the trastuzumab-IR700Dye conjugate led to a strong anti-cancer effect for HER2-positive cancer cells, including HER2 low-expressing cells, trastuzumab-resistant cells, and brain metastasis of HER2-positive breast cancer.

Our in vivo study also demonstrated that the HER2 Affibody-IR700Dye conjugate and the trastuzumab-Alexa488 conjugate bound to the HER2 protein and imaged only HER2-positive tumor (MDA-MB361) specifically. In whole-body images, IR700Dye showed a strong fluorescence intensity in the MDA-MB361 tumor, but Alexa488 could not be detected (Figure 7a). The reason is that IR700Dye allows deep signal penetration but the tissue penetration of Alexa488 is too poor to go through mouse skin. However, in the ex vivo study, the MDA-MB361 tumor showed a stronger fluorescence intensity of both IR700Dye and Alexa488 (Figure 7b). Therefore, these results confirm that both HER2 Affibody and trastuzumab bound to the HER2-positive tumor without competition. In addition, ex vivo fluorescence images showed a discrepancy in the fluorescence intensity between the HER2 Affibody-IR700Dye conjugate and the trastuzumab-Alexa488 conjugate in the liver and the kidney (Figure 8). Li et al. described that the extent of tissue distribution of protein therapeutics increases as the molecular size decreases, and kidneys represented the most abundant disposition site among all tissues, especially for smaller proteins [32]. Additionally, Mczyska et al. found an increased uptake of the IR700Dye-labeled Affibody conjugate in the kidney and suggested that it is associated with renal elimination and re-absorbance of the Affibody molecules [33]. Additionally, Seyed et al. showed that hydrophobic patches or positive charges in the proteins promote liver uptake using Affibody molecules [34]. These results are in agreement with our ex vivo results.

While this study showed that the combination of NIR-PIT using the Affibody-IR700Dye conjugate and the trastuzumab-IR700Dye conjugate is an attractive candidate for clinical use, we need further studies to examine the full therapeutic potential of this approach and also to know the effect of Affibody molecules remaining in the kidney and the liver for a long time and whether or not the Affibody-IR700Dye conjugate can actually cross the blood–brain barrier to treat brain metastases. However, Affibody and IR700Dye are already used clinically [35,36,37] (NCT03769506), and therefore, we would expect that the combination of NIR-PIT using the Affibody-IR700Dye conjugate and the trastuzumab-IR700Dye conjugate can be safely and rapidly translated into clinical practice. This study clearly demonstrated that the combination of NIR-PIT using the HER2 Affibody-IR700Dye conjugate and the trastuzumab-IR700Dye conjugate represents a new treatment strategy for heterogeneous HER2-positive cancer, including HER2 low-expressing cancer, trastuzumab-resistant cancer, and brain metastasis.

## 4. Materials and Methods

### 4.1. Cell Culture

The human breast cancer cell lines SK-BR3, MDA-MB361, MCF7, and JIMT1 were obtained from the American Type Culture Collection (SK-BR3, MDA-MB361, ATCC^®^, Manassas, VA, USA), the RIKEN Cell Bank (MCF7, Ibaraki, Japan), and the DSMZ (JIMT1, Braunschweig, Germany). MDA-MB361 was established from a brain metastasis of breast cancer. JIMT1 was established from a pleural effusion of breast cancer and is insensitive to HER-2-inhibiting drugs, e.g., trastuzumab (Herceptin).

The SK-BR3 cell line was cultured in McCoy’s 5A medium (GIBCO^®^, Life Technologies, Carlsbad, CA, USA) supplemented with 10% fetal bovine serum (FBS; GIBCO^®^, Life Technologies, Carlsbad, CA, USA) and 1% penicillin-streptomycin (Invitrogen, Life Technologies, Carlsbad, CA, USA). Other cell lines were cultured in DMEM (GIBCO^®^, Life Technologies, Carlsbad, CA, USA) supplemented with 10% fetal bovine serum and 1% penicillin-streptomycin. All cell lines were kept in a humidified environment containing 5% CO_2_ at 37 °C. The medium was changed every other day.

### 4.2. Immunocytochemistry (ICC)

Prior to ICC, coverslips needed to be put at the bottom of the wells in a 24-well plate. In total, 1 × 10^5^ HER2-positive breast cancer cells (SK-BR3, MDA-MB361, and JIMT1) and HER2-negative breast cancer cells (MCF7) were seeded on the coverslips. Cells were fixed in 4% paraformaldehyde for 15 min and washed with phosphate-buffered saline (PBS) twice. Nonspecific sites were then blocked with 3% bovine serum albumin (BSA, Sigma-Aldrich, Burlington, MA, USA) in PBS for 30 min at room temperature. Cells were incubated with anti-HER2 antibody (HER2/ErbB2 (D8F12) XP TM rabbit mAb, Cell Signaling Technology, Danvers, MA, USA) overnight at 4 °C and washed with PBS, followed by incubation with the appropriate Alexa Fluor 488 secondary antibody (1:1000, anti-rabbit IgG Fab2, Alexa Fluor ^®^ 488, Cell Signaling Technology, Danvers, MA, USA) for 1 h at room temperature. The coverslips were added to the mounting medium with DAPI (Vectashield Mounting Medium with DAPI, Tokyo, Japan) for 10 min and were observed using a fluorescence microscope (LSM 700 confocal, ZEISS, Oberkochen, Germany).

### 4.3. Western Blotting Analysis

The general procedure for Western blot analysis was performed as follows. Cells (SK-BR3, MDA-MB361, JIMT1, and MCF7) in a 60-mm dish were washed with ice-cold PBS (-) and 100 µL of modified RIPA buffer (Thermo Fisher Scientific, Waltham, MA, USA) containing a protease inhibitor tablet (cOmplete™, Mini Protease Inhibitor Cocktail, Roche, Mannheim, Germany), 1% ethylenediaminetetraacetic acid (EDTA), and 1% protease/phosphatase inhibitor (Thermo Fisher Scientific, Waltham, MA, USA) was added. The cells were scraped from the dish and centrifuged at a high speed at 4 °C for 4 min. The supernatant was transferred to fresh tubes and the protein concentration was determined using a Qubit Protein Assay Kit (Thermo Fisher Scientific, Waltham, MA, USA) according to the manufacturing protocol described. The samples were then heated for 5 min at 100 °C and equal amounts of proteins (30 µg) were subjected to SDS-PAGE. The proteins were transferred to a polyvinylidene fluoride (PVDF) membrane. After blocking with 5% non-fat milk, the membrane was incubated with primary antibody (HER2/ErbB2 (D8F12) XPTM Rabbit mAb, Cell Signaling Technology, Danvers, MA, USA) at 4 °C overnight. Subsequently, the membrane was incubated with secondary antibodies (EasyBlot anti-rabbit IgG (HRP), Gene Tex, Inc., Alton Pkwy Irvine, CA, USA). As a loading control, the membrane was also subjected to immunoblotting using β-actin polyclonal Ab (beta-actin antibody, Gene Tex, Inc., Alton Pkwy Irvine, CA, USA) as a primary antibody and EasyBlot anti-rabbit IgG (HRP) (Gene Tex, Inc., Alton Pkwy Irvine, CA, USA) as a secondary antibody. The immunoreactive bands were visualized with chemiluminescence using ECL Western blotting detection reagent (ECL Prime Western Blotting Detection Reagent, GE Health Care Amersham^TM^, Chicago, IL, USA). Image Quant LAS-500 (GE Healthcare, Chicago, IL, USA) was used for visual assessment.

### 4.4. HER2 Affibody-IR700Dye Conjugate, Trastuzumab-IR700Dye or Alexa488 Conjugate

HER2 Affibody (Affibody AB, Solna, Sweden) was dissolved in PBS to a final concentration of 1 mg/mL and dithiothreitol (DTT) was added to a final concentration of 20 mM at >pH 7.5. After incubation at room temperature for 2 h, excess DTT was removed from the conjugate by passage through a NAP5 column (GE Healthcare, Chicago, IL, USA). Then, the HER2 Affibody was incubated with a 5-fold molar excess of IRDye700DX–maleimide (MW: 1979.23, LI-COR Biosciences, Lincoln, NE, USA) for 2 h at 37 °C. After conjugation, the solution was applied to protein desalting spin columns (Thermo Fisher Scientific, Waltham, MA, USA) and centrifuged at 1500× *g* for 2 min to purify the sample. To produce the trastuzumab conjugate, trastuzumab (MW: 146 kDa, BioVision, Milpitas, CA, USA) was incubated with a 5-fold molar excess of IR700DX-NHS ester (MW: 1954.22, RAKUTEN medical, San Mateo, CA, USA) in PBS at 37 °C for 1 h. Then, the mixture was purified with a protein desalting spin column (Thermo Fisher Scientific, Waltham, MA, USA). Additionally, trastuzumab was incubated with a 5-fold molar excess of Alexa488 (MW: 643.41, Thermo Fisher Scientific, Waltham, MA, USA) and purified in the same manner as above.

### 4.5. Confocal Microscopy Imaging of HER2 Affibody-IR700Dye and Trastuzumab-Alexa488 Conjugates

HER2-positive breast cancer cells (SK-BR3, MDA-MB361, and JIMT1) and HER2-negative breast cancer cells (MCF7) were seeded on a glass-bottom 96-well plate. To test the specificity of the conjugate binding, the HER2 Affibody-IR700Dye conjugate (1 µM) and the trastuzumab-Alexa488 (1 µM) conjugate were added to the media and the cells were incubated for 30 min at 37 °C. After washing the cells with PBS, the cells were examined using a BZ-X800 fluorescence microscope (Keyence, Osaka, Japan). To overlay images, ImageJ was used [38].

### 4.6. Flow Cytometry

After detaching the cells with trypsin/ethylenediaminetetraacetic acid (EDTA), to a 1-milliliter cell suspension including 1 × 10^6^ cells, HER2 Affibody-IR700Dye conjugate (1 µM) and trastuzumab-Alexa488 (1 µM) conjugate were added. The cell suspension was incubated for 30 min at 37 °C, washed with PBS, and then, the fluorescence intensity was examined using a flow cytometer (CytoFLEX, Beckman Coulter Life Sciences, Inc., Brea, CA, USA).

### 4.7. Cell Viability Assay

The cell viability was determined based on fluorescence intensity using an alamarBlue assay. Briefly, cells were seeded at 1 × 10^4^/well in flat-bottom 96-well culture plates and allowed to grow for 24 h, followed by incubation with HER2 Affibody-IR700Dye conjugate and/or trastuzumab-IR700Dye conjugate (0–0.3 µM) for 2 h at 37 °C. After washing the cells with PBS, near-infrared light (0–50 J/cm^2^) was irradiated from the bottom of the wells. After near-infrared (NIR) light irradiation, the cells were incubated with alamarBlue solution (10 µL/100 µL in medium) for 2 h and the fluorescence intensity was measured at 540–570/580–610 nm using a microplate reader (Power Scan’MX, DS PHARMA BIOMEDICAL, Osaka, Japan). The cell viabilities were followed for 5 days after NIR light irradiation. The results of the experiments are presented as the mean ± standard deviation (SD), which were performed in at least three wells per sample and repeated more than three times. The cell viabilities 24 h after the NIR-PIT were analyzed using a one-way ANOVA with Tukey–Kramer post hoc tests, as shown in Figure 6 (* *p* < 0.05; ** *p* < 0.01).

### 4.8. Cell Images before and after Near-Infrared (NIR) Light Irradiation

Cells were seeded at 1 × 10^4^/well in glass-bottom 96-well culture plates and allowed to grow for 24 h, followed by incubation with HER2 Affibody-IR700Dye conjugate (0.3 µM) or trastuzumab-IR700Dye conjugate (0.3 µM) for 2 h at 37 °C. After washing the cells with PBS, near-infrared light (50 J/cm^2^) was irradiated from the bottom of the wells. Images of the cells were taken by a microscope (LSM confocal, ZEISS, Oberkochen, Germany) before and after NIR irradiation.

### 4.9. Cell Apoptosis/Necrosis Assay

Cells (SK-BR3, MDA-MB361, JIMT1, and MCF7) were seeded at 1 × 10^4^/well in glass-bottom 96-well culture plates and allowed to grow for 24 h, followed by incubation with HER2 Affibody-IR700Dye conjugate (0.3 µM) or trastuzumab-IR700Dye conjugate (0.3 µM) for 2 h at 37 °C. After washing the cells with PBS, near-infrared (NIR) light (50 J/cm^2^) was irradiated from the bottom of wells. Then, apoptosis or necrosis of the cells was determined using an Apoptosis/Necrosis Assay Kit (ab176749, Abcam, Cambridge, UK) as per the manufacturing protocol described.

### 4.10. Near-Infrared Photoimmunotherapy (NIR-PIT) Illuminator

Our own designed NIR-PIT illuminator was used for all NIR-PIT experiments, with the same settings as previously described [16]. The NIR-PIT illuminator is composed of 8 light-emitting diodes (LED: SMBB690D-1100-02 × 8, EPITEX, Inc., Kyoto, Japan), whose peak wavelength of emission is 690 nm. In this study, the power density of the LEDs was set to 200 mW/cm^2^ at 500 mA.

### 4.11. Animal Imaging

Four-week-old female athymic mice (BALB/cSlc-nu/nu, Japan SLC, Shizuoka, Japan; *n* = 3, body weight 12–17 g) were used in the animal studies. The mice were acclimatized for 1 week and housed under specific-pathogen-free (SPF) conditions with a 12-h light/ dark cycle in cages. They were given free access to feed (D10001, AIN-76A, Research Diet Inc., New Brunswick, NJ, USA) and sterilized water before the experiments started. The MDA-MB361 model (*n* = 3) and the MCF7 model (*n* = 3) of breast cancer were established in mice by injecting 1 × 10^6^–10^7^ cells suspended in 200 µL of a 1:1 mixture of Matrigel (Becton Dickinson, Tokyo, Japan) and PBS subcutaneously into the shoulder (MDA-MB361) and dorsum (MCF7). The experiments were performed 4 weeks after the injection. HER2 Affibody-IR700Dye conjugate and trastuzumab-Alexa488 conjugate were mixed and injected into the tail vein in an amount of 100 µL. After 24 h, the mice were humanely killed by an overdose of pentobarbital via intraperitoneal injection. The whole mice bodies and removed tissues (tumor, heart, liver, kidney, stomach, spleen, intestine, and muscle) were imaged using the IVIS Lumina iii In Vivo Imaging System (PerkinElmer, Waltham, MA, USA).

### 4.12. Immunohistochemistry

Excised tumors were fixed in 10% formaldehyde solution overnight and were embedded in a paraffin block. From the paraffin-embedded tissues of both HER2-positive tumors (MDA-MB361) and HER2-negative tumors (MCF7), serial sections of 3 μm in thickness were prepared. After deparaffinization, the sections were incubated with Immunosaver (Nissin EM, Tokyo, Japan) at 98 °C for 45 min to retrieve the antigen and then treated with ethanol containing 0.3% H_2_O_2_ to block endogenous peroxidase. The sections were incubated with 5% normal goat serum (Agilent Technologies Inc., Santa Clara, CA, USA) for 10 min at room temperature and then incubated with the primary antibody against HER2 (HER2/ErbB2, clone D8F12, 1:400, Cell Signaling Technology, Danvers, MA, USA) for 60 min at room temperature. Then, the sections were treated with secondary antibodies (Histofine Simple Stain MAXPO MULTI; Nichirei Bioscience Inc., Tokyo, Japan) for 30 min at room temperature. The color was developed using 3,3′-diaminobenzidine·4HCl (DAB Substrate Kit; Nichirei Bioscience Inc.). After nuclear staining with hematoxylin, the slides were observed under a light microscope (BX53, Olympus, Tokyo, Japan).

All animals were treated in accordance with the Ethical Guidelines for Investigations of Experimental Animals of the Nippon Dental University School of Life Dentistry at Niigata (No. 219).

## 5. Conclusions

The combination of NIR-PIT using the HER2 Affibody-IR700Dye conjugate and the trastuzumab-IR700Dye conjugate enhanced the cytotoxic effect for HER2-positive breast cancer cells, including HER2 low-expressing cancer cells, trastuzumab-resistant cancer cells, and brain metastatic cells. This approach has the potential to improve the efficiency of current NIR-PIT, especially for heterogeneous HER2-positive cancer.

## Data Availability

A phase III human clinical trial of NIR-PIT is currently underway. (https://clinicaltrials.gov/ct2/show/NCT03769506, accessed on 15 October 2021).

## Supplementary Materials

The following are available online at https://www.mdpi.com/article/10.3390/ijms222212213/s1.

## Abbreviations

HER2	Human epidermal growth factor receptor type 2
EGFR	Epidermal growth factor receptor
NIR-PIT	Near-infrared photoimmunotherapy
mAb	Monoclonal antibody
ICC	Immunocytochemistry
