# Supplementary material for: Combination of Near-Infrared Photoimmunotherapy Using Trastuzumab and Small Protein Mimetic for HER2-Positive Breast Cancer
Source: Int J Mol Sci. 2021 Nov 11;22(22):12213. doi: 10.3390/ijms222212213 (PMC8618566; doi:10.3390/ijms222212213)
Supplement: Supplementary file 1 [file ijms-22-12213-s001.zip › ijms-1453010-supplementary.pdf]

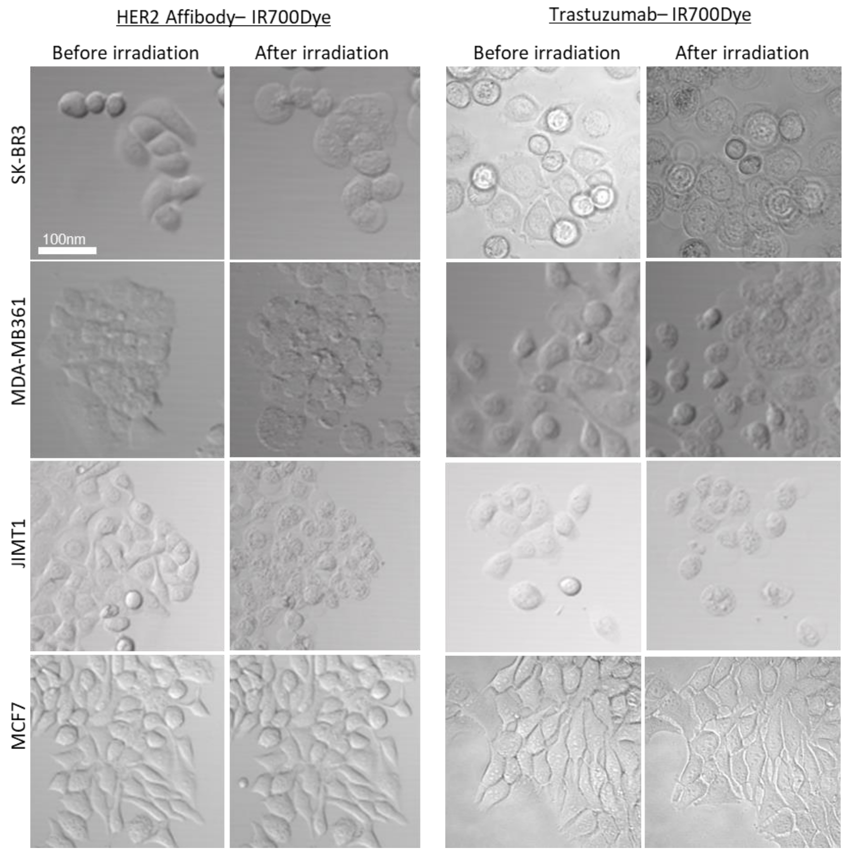

Supplementary Information Figure 1. Cell images before and after NIR-PIT.

Cell images before and after NIR-PIT using HER2 Affibody-IR700Dye conjugate or Trastuzumab-IR700Dye conjugate. Images of cancer cells showed that only HER2-positive cancer cells (SK-BR3, MDA-MB361, JIMT1) displayed morphological evidence of cellular bursting and/or bleb formation, while the morphology of HER2-negative cancer cells (MCF7) remained unchanged. Scale bar: 100 µm.

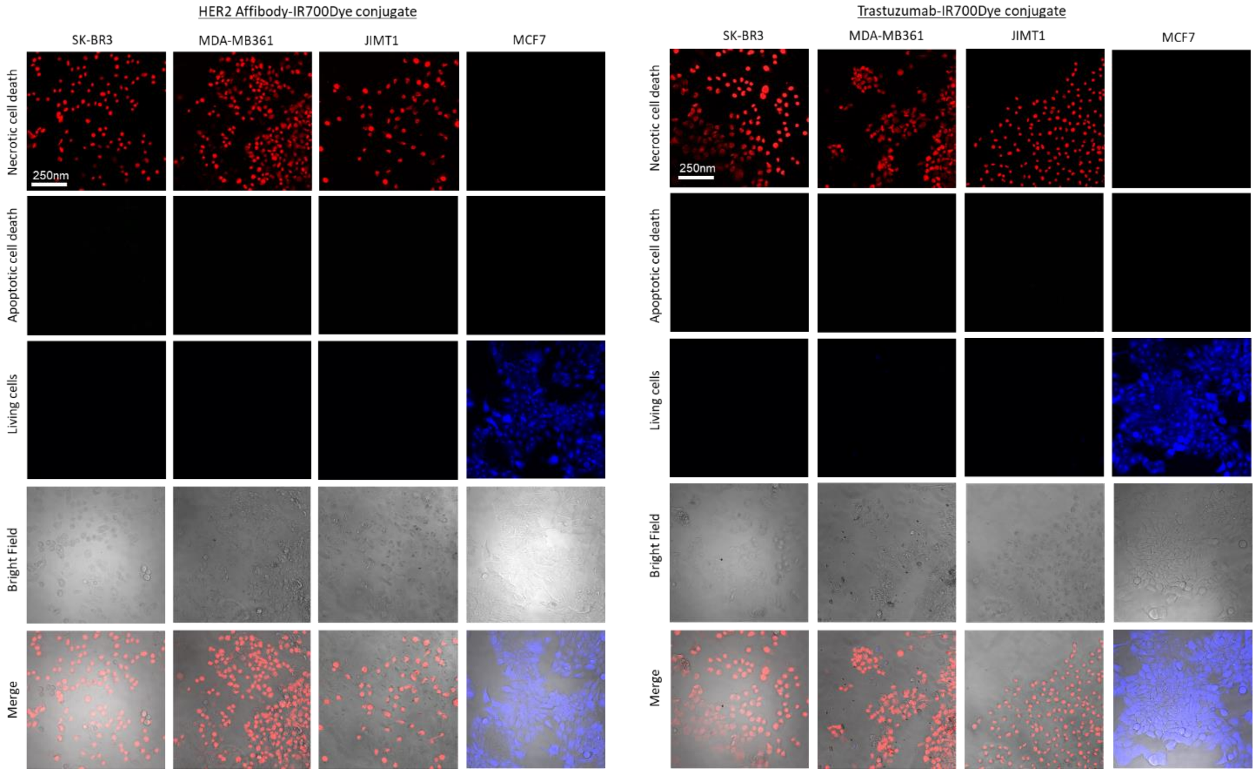

Supplementary Information Figure 2. Apoptosis/Necrosis Assay.

Apoptosis/Necrosis Assay Kit stains living cells blue, apoptotic dead cells green, and necrotic dead cells red. HER2-positive cancer cells (SK-BR3, MDA-MB361 and JIMT1) exposed to NIR-PIT by either HER2 Affibody-IR700Dye conjugate or Trastuzumab-IR700Dye showed red signal whereas HER2-negative cancer cells (MCF7) stained blue. Scale bar: 250 µm.

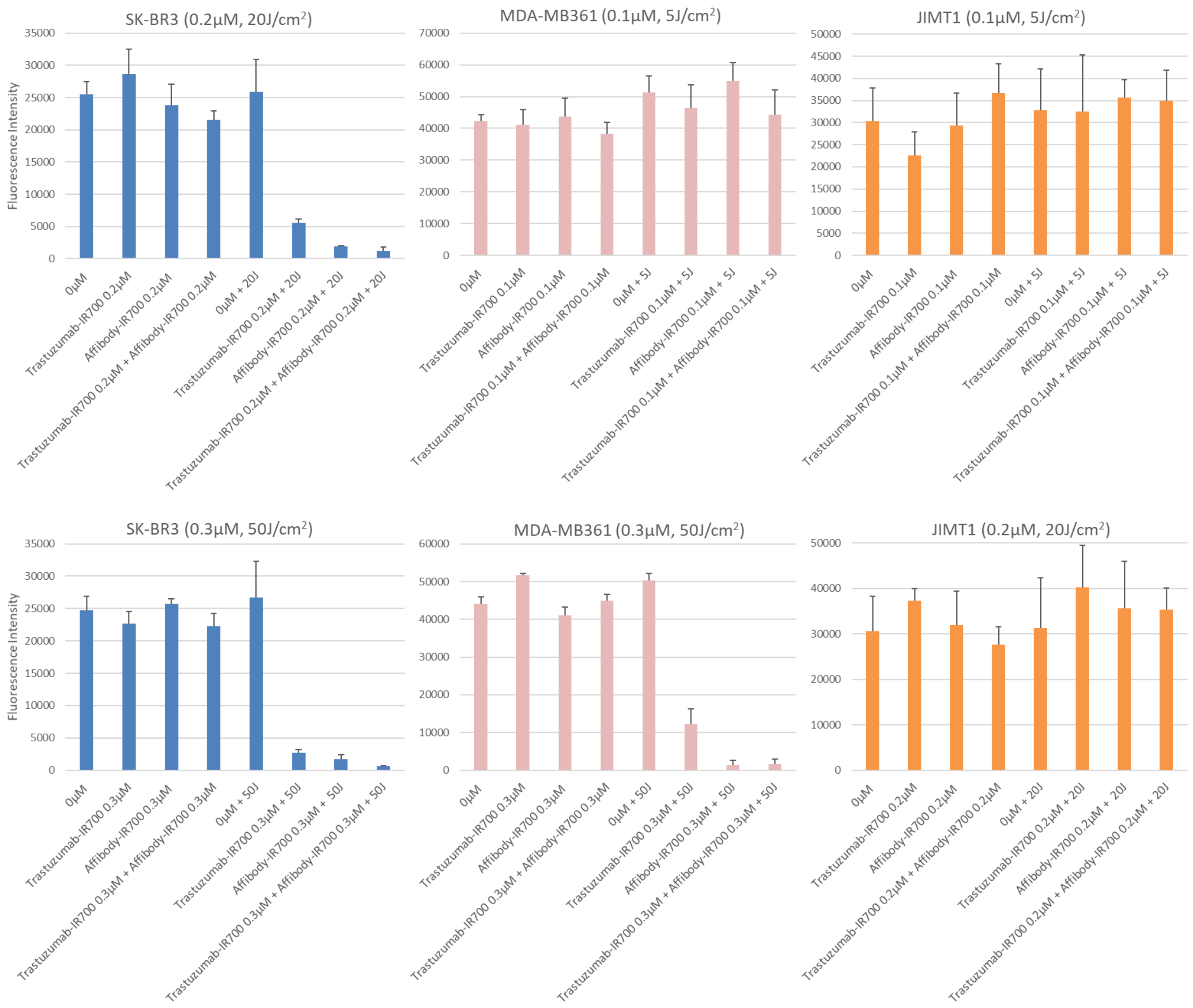

Supplementary Information Figure 3. Cell viability after NIR-PIT.

HER2-positive cancer cells (SK-BR3, MDA-MB361 and JIMT1) and HER2-negative cancer cells (MCF7) were incubated with HER2 Affibody-IR700Dye conjugate and/or Trastuzumab-IR700Dye conjugate (0, 0.1, 0.2, 0.3µM) and irradiated near infrared light (690nm) (0, 20, 50J/cm^{2}). SK-BR3 cells were killed by NIR-PIT with more than 0.1µM conjugate and 5J/cm^{2} irradiation, but MDA-MB361 cells need more than 0.2µM conjugate and 20J/cm^{2} irradiation, and JIMT1 cells need more than 0.3µM conjugate and 50J/cm^{2} irradiation to be affected. The effect of NIR-PIT depends on the level of HER2 expression, the concentration of the conjugates, and the power of irradiation. (n = 6). Data are presented as means ± SD.
